# Navigating tradition and treatment: cultural identity, social expectations, and type 1 diabetes self-management among young Saudi adults–a qualitative narrative inquiry

**DOI:** 10.3389/fpubh.2026.1920664

**Published:** 2026-08-31

**Authors:** Abdulaziz M. Alodhailah, Mohammed Almutairi, Ahmed Alsadoun, Raeed Alanazi, Zainab Alfar, Thurayya Eid, Waleed M. Alshehri

**Affiliations:** 1 Department of Medical-Surgical Nursing, College of Nursing, King Saud University, Riyadh, Saudi Arabia; 2 Department of Nursing Administration and Education, College of Nursing, King Saud University, Riyadh, Saudi Arabia

**Keywords:** chronic illness stigma, cultural identity, narrative inquiry, Ramadan fasting, Saudi Arabia, self-management, type 1 diabetes mellitus, young adults

## Abstract

**Background:**

Type 1 diabetes (T1D) requires lifelong, intensive self-management that intersects with cultural practice in collectivistic societies, where communal food traditions, religious observance, and family expectations shape daily routines. Despite an estimated incidence of 18–20 per 100,000 annually in Saudi Arabia, qualitative evidence examining how young adults navigate disease management alongside cultural identity remains limited.

**Objective:**

This study explored how young Saudi adults with T1D experience and negotiate the interplay between cultural traditions, social expectations, and diabetes self-management in everyday life.

**Methods:**

A qualitative narrative inquiry design was employed. Fourteen Saudi adults (aged 18–30 years) with a confirmed T1D diagnosis of at least 1 year were recruited through purposive and snowball sampling. Semi-structured interviews were conducted in Arabic, translated using a forward-backward protocol, and analyzed through reflexive thematic analysis. Reporting followed the Consolidated Criteria for Reporting Qualitative Research (COREQ).

**Results:**

Five interconnected themes emerged: (1) the invisible struggle of managing a hidden condition; (2) cultural food negotiations within social contexts; (3) Ramadan as a spiritual and medical challenge; (4) social camouflage and disclosure dilemmas; and (5) resilience through cultural innovation. Three narrative identity patterns, namely Journey, Warrior, and Bridge-Builder, further characterized how participants made meaning of their experiences. Deviant cases revealed family conflict, unsuccessful fasting attempts, and persistent concealment that participants described as harmful to their self-management.

**Conclusion:**

Young Saudi adults with T1D exercise genuine cultural agency in integrating traditional practices with biomedical management. These findings support culturally responsive care models that treat cultural knowledge as a clinical resource rather than a barrier, and point to the need for family-inclusive diabetes education and collaborative pre-Ramadan planning. Future research should use longitudinal designs, incorporate clinical outcome measures, and extend inquiry to rural Saudi and Gulf Cooperation Council populations.

## 1 Introduction

Type 1 diabetes mellitus (T1D) is an autoimmune condition requiring lifelong intensive self-management. In Saudi Arabia, T1D incidence is estimated at 18–20 per 100,000 persons annually, with the highest rates among children and young adults (1). Self-management encompasses continuous glucose monitoring, insulin therapy, dietary regulation, and sustained lifestyle adaptation, demands that carry substantial physical, psychological, and social consequences (2). For young adults aged 18–30 years, this burden coincides with a developmentally critical transition period in which individuals must simultaneously establish educational and professional trajectories, build interpersonal relationships, and consolidate cultural identities (3). The transfer from pediatric to adult diabetes care is consistently identified as a period of heightened vulnerability, characterized by deteriorating glycemic control and elevated risk of acute and long-term complications (4, 5). These adverse outcomes reflect reduced family supervision, irregular lifestyle patterns, and the difficulty of engaging with adult healthcare systems not fully attuned to young adults’ developmental needs (4, 5).

The Saudi Arabian sociocultural context introduces distinctive considerations for T1D self-management. Traditional practices surrounding hospitality, communal dining, and religious observance center on food consumption patterns that can directly conflict with clinical dietary recommendations (6). Young adults with T1D must negotiate between deeply held cultural traditions and medical requirements, often without adequate support from social networks that may lack understanding of the condition’s demands (7, 8). This tension can be understood as a form of “living between cultures”: a dynamic process of reconciling traditional cultural practices with biomedical management imperatives. Drawing analogically on Berry’s (9) acculturation framework, not to describe migration-related cultural change but to illuminate how individuals integrate two competing normative systems into a coherent way of living, this study foregrounds the cultural negotiation at the heart of T1D self-management (9). These tensions are particularly pronounced for young Saudi adults who are simultaneously establishing independence, building careers, and maintaining family relationships within a collectivistic social structure (6).

Religious practices and psychosocial factors further shape the T1D experience in Saudi Arabia. Islamic principles emphasizing communal solidarity, family responsibility for health, and trust in divine providence alongside biomedical treatment can both facilitate and complicate self-management, depending on how families interpret and apply these values (10, 11). Ramadan fasting is a prominent example (12). Although evidence-based clinical guidelines address the physiological risks of altered eating and medication timing (13, 14), the subjective experience of negotiating this central religious obligation alongside medical requirements has received limited scholarly attention (13, 14). Beyond religious observance, the psychosocial dimensions of T1D in collectivistic contexts include concerns about social stigma, marriageability, and family-level reputation (15, 16). These challenges directly affect treatment adherence, glycemic control, and quality of life, pointing to the need for diabetes care in Saudi Arabia that extends well beyond clinical skill transfer (17–19).

Despite growing T1D prevalence among young Saudi adults, the existing literature remains predominantly quantitative, focused on clinical protocols and physiological outcomes (1, 20, 21). Qualitative studies addressing Saudi adults’ diabetes experiences are scarce; those that exist have focused mainly on Type 2 diabetes, offering little insight into the distinct developmental and sociocultural challenges facing young adults with T1D (6, 20, 21). This evidence gap risks perpetuating care models that position cultural practices as obstacles. The present study addresses this gap through narrative inquiry, centering participant voices. The guiding research question was: How do young Saudi adults with T1D experience and navigate the intersection of cultural practices, social expectations, and diabetes self-management in their daily lives? The study aims to generate insights that can inform culturally responsive, person-centered diabetes care in Saudi Arabia.

## 2 Materials and methods

### 2.1 Study design and theoretical framework

A narrative inquiry design (22) was employed to explore how young Saudi adults construct and make meaning of their T1D experiences within their cultural context. Narrative inquiry was selected because it centers participants’ storytelling, providing rich insight into how individuals integrate medical and cultural identities over time. Data were analyzed using Braun and Clarke’s (23) reflexive thematic analysis, chosen for its epistemological flexibility and its capacity to identify patterns across multiple narratives while preserving attention to individual stories. Narrative inquiry provided the methodological framework for exploring how young adults with Type 1 diabetes constructed meaning around their illness experiences, cultural identity, family relationships, and religious beliefs over time. The interview process encouraged participants to share personal stories, including experiences before diagnosis, adaptation following diagnosis, interactions with family and healthcare providers, and the development of self-management identities. Reflexive thematic analysis, following Braun and Clarke’s approach, was used as the analytical method to identify shared patterns of meaning across participants’ narratives while maintaining sensitivity to the individual contexts and temporal structures of each story. Rather than treating narratives as isolated accounts, analysis focused on how participants constructed identities and negotiated cultural expectations through their diabetes experiences.

The theoretical framework drew on cultural psychology perspectives recognizing culture as a constitutive element of human experience rather than simply a background factor (24). This orientation allowed the research to examine not only what participants experienced but how they interpreted those experiences through cultural meaning-making frameworks.

### 2.2 Participant recruitment and selection

Participants were recruited using a purposive sampling strategy to capture diverse experiences while maintaining focus on the research question. Recruitment occurred through professional networks, family connections, and university student services. Snowball sampling supplemented initial recruitment, with early participants referring potentially eligible individuals within their networks. No prior clinical, supervisory, or personal relationship existed between the lead researcher and any participant prior to recruitment. Potential participants were screened for eligibility before being approached, and participation was entirely voluntary.

Inclusion criteria were: (i) Saudi nationality; (ii) age 18–30 years, capturing the transition-to-adulthood period identified as a time of heightened T1D vulnerability (3–5); (iii) T1D diagnosis confirmed by medical records for at least 1 year, ensuring sufficient management experience for meaningful narrative reflection; (iv) capacity to provide written informed consent; and (v) fluency in Arabic.

Exclusion criteria were: (i) significant cognitive impairment precluding meaningful participation; (ii) severe psychiatric illness requiring active inpatient treatment; and (iii) concurrent major medical conditions that could confound the diabetes experience.

The final sample comprised 14 participants [8 males (57.1%), 6 females (42.9%)] aged 19–28 years (mean 23.1 years, SD 2.9). T1D duration ranged from 2 to 15 years (mean 8.4 years, SD 4.2). Eight participants were current university students, 3 were university graduates, and 3 held secondary-level education. Regarding employment, 4 worked full time, 4 part-time, 4 were students without paid work, and 2 were seeking employment. Detailed individual characteristics are presented in Table 1.

**Table 1:** Participant characteristics (*N* = 14).

Pseudonym	Gender	Age (years)	Age at diagnosis (years)	T1D duration (years)	Insulin regimen	Glucose Monitoring	Ramadan fasting (last year)	Educational background	Work/study status	Marital status
P1	Male	19	14	5	MDI	Flash	Yes	University student	Student (not employed)	Single
P2	Female	21	9	12	CSII	CGM	Partial	University student	Part-time	Single
P3	Male	23	8	15	CSII	CGM	Yes	University graduate	Full-time	Married
P4	Female	24	14	10	MDI	Flash	Yes	University student	Student (not employed)	Single
P5	Male	20	18	2	MDI	No	No	University student	Student (not employed)	Single
P6	Female	26	11	15	CSII	CGM	Partial	University graduate	Full-time	Married
P7	Male	22	10	12	MDI	Flash	Yes	University student	Part-time	Single
P8	Female	25	19	6	MDI	Flash	Yes	University graduate	Full-time	Engaged
P9	Male	28	16	12	CSII	CGM	Yes	Secondary education	Full-time	Married
P10	Female	22	7	15	CSII	CGM	Partial	University student	Part-time	Single
P11	Male	19	15	4	MDI	Flash	No	Secondary education	Unemployed	Single
P12	Female	27	12	15	MDI	Flash	Yes	University student	Student (not employed)	Single
P13	Male	24	11	13	CSII	CGM	Yes	Secondary education	Unemployed	Single
P14	Male	23	17	6	MDI	No	Partial	University student	Part-time	Single

MDI, Multiple Daily Injections; CSII, Continuous Subcutaneous Insulin Infusion (insulin pump); CGM, continuous glucose monitoring; Flash, Flash Glucose Monitoring (e.g., FreeStyle Libre). Ramadan Fasting: Yes, fasted ≥20 days; Partial = fasted <20 days; No = did not fast. T1D Duration = current age minus age at diagnosis.

### 2.3 Ethical considerations

The study was conducted in full accordance with the ethical principles of the Declaration of Helsinki. Ethical approval was granted by the Institutional Review Board of King Saud University, Saudi Arabia (Reference: KSU-HE-25-954; approved 26 August 2024). All participants provided written informed consent after receiving comprehensive information regarding the study’s purposes, procedures, potential risks and benefits, voluntariness, right to withdraw without consequence, and data confidentiality measures. To protect anonymity, participants were assigned pseudonyms (P1–P14), and all data were stored in password-protected files accessible only to the research team.

Given the sensitive nature of discussing chronic illness, care was taken to ensure participants understood their right to decline any question or end the interview at any time without consequence. Contact information for diabetes support services and mental health resources was provided to all participants. No adverse events occurred during the study, and several participants expressed appreciation for the opportunity to share their experiences.

### 2.4 Data collection procedures

Semi-structured interviews were conducted between October 2024 and January 2025 using a researcher-developed interview guide consisting of 15 open-ended questions (Appendix A). The guide was informed by the relevant literature and study objectives and was refined following pilot testing with two individuals who were not included in the final sample. Questions were designed to facilitate in-depth narratives rather than brief responses, encouraging participants to describe their lived experiences of managing diabetes within their cultural and social contexts.

Interviews were conducted in Arabic at locations selected by participants to promote comfort, privacy, and openness, including offices, and private rooms. Interviews were conducted by A. M. A., a PhD nurse with clinical experience and formal training in qualitative research methods and interviewing techniques. The interviewer had no prior clinical or professional relationship with any participant, minimizing potential influence on participants’ responses.

Interview duration ranged from 45 to 90 min (mean: 67 min), determined by response richness and natural narrative conclusion. All interviews were audio-recorded on encrypted digital devices with participant permission. Field notes were maintained throughout data collection, documenting contextual information, nonverbal observations, and preliminary analytical reflections.

Data collection and analysis occurred concurrently: following each interview, transcripts were reviewed and coded before subsequent interviews were conducted. Recruitment continued until additional interviews no longer generated new concepts, narrative structures, or meaningful variations in participants’ experiences; saturation was determined collectively through regular discussions among the research team. Data collection continued until thematic saturation was reached. No new themes or substantial variations emerged after interview 12; interviews 13 and 14 confirmed saturation.

### 2.5 Translation procedures

All interviews were conducted and transcribed verbatim in Arabic. Data analysis was initially conducted using the original Arabic transcripts to preserve contextual meaning and minimise potential loss of culturally embedded information. For reporting purposes, selected participant quotations were translated into English by the research team, who is bilingual in Arabic and English and has expertise in qualitative nursing research.

A forward-backward translation protocol was employed to ensure linguistic accuracy and conceptual equivalence. Initially, the Arabic transcripts were translated into English by the researchers (forward translation). An independent bilingual researcher, who was not involved in the initial translation process, then performed a backward translation of a randomly selected sample of four transcripts (29% of the total dataset) from English into Arabic. The original Arabic transcripts, forward translations, and back-translations were compared, and any discrepancies were discussed and resolved collaboratively by the research team.

In addition to linguistic accuracy, attention was given to the readability and interpretability of translated quotations. Translations were refined to ensure that participants’ narratives were expressed naturally in English while maintaining the original meaning, emotional tone, and cultural context. Where direct translation resulted in unclear or unfamiliar expressions for English-speaking readers, minor linguistic adaptations were made to improve clarity and flow without altering the underlying meaning. Interpretive decisions were documented and discussed among bilingual members of the research team to ensure that translated accounts remained faithful to participants’ experiences while being accessible and understandable to an international readership.

### 2.6 Data analysis

Data analysis followed Braun and Clarke’s reflexive thematic analysis (23), implemented iteratively rather than linearly to ensure analytical depth and rigor. Phase 1 (familiarization) involved repeated reading of transcripts alongside audio recordings, with initial impressions recorded in analytical memos. Phase 2 (coding) generated inductive codes capturing both semantic and latent meanings, with close attention to linguistic nuances and narrative structures. Phase 3 (theme development) clustered codes into candidate themes, which were then tested against the raw data. Phase 4 (theme review) refined themes through iterative team discussion and cross-referencing with both coded extracts and the full dataset. Phase 5 (theme definition) established precise conceptual boundaries and descriptive names for each theme. Phase 6 (report production) constructed a coherent analytical narrative directly addressing the research question. All analytical decisions were documented in an audit trail. An inductive analytic approach was adopted, allowing codes, themes, and narrative identity patterns to emerge directly from participants’ accounts through iterative engagement with the data. Two members of the research team independently reviewed each transcript during the initial familiarisation stage. Coding was conducted iteratively using reflexive thematic analysis. Regular meetings were held throughout analysis to discuss coding decisions, refine theme development, and ensure interpretive coherence. Differences in interpretation were resolved through collaborative discussion rather than quantitative agreement measures, consistent with reflexive thematic analysis.

### 2.7 Trustworthiness and rigor

Multiple strategies were employed to enhance the trustworthiness and rigor of the findings. Prolonged engagement was achieved through extended interviews and follow-up contact when clarification was needed. Peer debriefing occurred regularly with research team members experienced in qualitative research but not directly involved in data collection.

Member checking was conducted with five participants who reviewed theme summaries and provided feedback on interpretive accuracy. Negative case analysis was used to identify experiences that challenged emerging themes, prompting refinement and qualification of findings. A detailed audit trail documented all analytical decisions and theme development processes.

Reflexivity was maintained throughout the research process through regular journaling about researcher assumptions, biases, and emotional responses to participant narratives. The lead researcher’s position as a healthcare professional with experience working with individuals with diabetes was acknowledged as both a potential asset in understanding medical aspects of participants’ experiences and a potential bias requiring ongoing reflection. The research team included members from nursing, public health, and cultural studies backgrounds, providing diverse interpretive perspectives. These reflexive practices, together with the peer debriefing and negative case analysis described above, served as explicit strategies to minimise interpretive bias. Reporting adhered to the Consolidated Criteria for Reporting Qualitative Research (COREQ; see Supplementary File S1).

## 3 Results

### 3.1 Participant characteristics

The 14 participants reflected diverse backgrounds within the target demographic. Ages ranged from 19 to 28 years, and T1D duration from 2 to 15 years, capturing perspectives from those recently diagnosed and individuals with extensive management experience. Participants were drawn from different urban areas; family structures varied, though all maintained close family relationships consistent with Saudi cultural norms. Several were married or engaged. All participants identified as Muslim. Full participant characteristics are presented in Table 1.

### 3.2 Overview of themes

Five major themes emerged from the narrative analysis, each encompassing multiple subthemes and representing different dimensions of participants’ experiences navigating T1D within their cultural context. These themes were interconnected rather than discrete, with participants’ narratives often weaving together elements from multiple themes. Table 2 summarizes the identified themes and subthemes.

**Table 2:** Themes and subthemes.

Theme	Subthemes	Brief description
1. The invisible struggle in managing a hidden chronic condition	1a. Hidden complexity of daily management tasks	The continuous, unobservable work of monitoring, calculating, and adjusting that constitutes daily T1D care
	1b. Challenges communicating needs to others	Difficulty explaining the nature and severity of T1D to family, peers, employers, and the public
	1c. Pressure to maintain normal appearances	Social expectation to appear healthy and unaffected, driving concealment behaviors
	1d. Emotional burden of constant self-surveillance	Mental exhaustion, hypervigilance, and chronic stress from unrelenting self-monitoring
2. Cultural food negotiations in balancing tradition and medical needs	2a. Adapting traditional recipes through ingredient substitution	Modifying cooking methods and ingredients to reduce glycemic impact while preserving cultural flavor
	2b. Navigating family expectations around food consumption	Managing pressure to eat traditional portions and dishes at family gatherings
	2c. Managing portion control in communal dining	Strategies for limiting intake without drawing attention during shared meals
	2d. Carbohydrate counting for traditional dishes	Developing specialized knowledge to estimate carbohydrates in culturally specific foods
	2e. Creating new traditions honoring heritage and health	Introducing diabetes-friendly alternatives that become family favorites
3. Ramadan as a test of faith and health through spiritual and medical integration	3a. Spiritual significance of fasting versus health concerns	The tension between religious duty and medical risk, and how participants reconciled both
	3b. Medical preparations for safe participation	Pre-Ramadan consultations, insulin adjustments, meal planning, and emergency protocols
	3c. Family and community support during Ramadan	Roles of family members in monitoring health, preparing appropriate meals, and providing encouragement
	3d. Protocols for breaking fasts during emergencies	Predetermined thresholds and plans for safely ending a fast when glucose levels became dangerous
	3e. Emotional satisfaction of successful religious participation	Feelings of accomplishment, spiritual fulfillment, and social belonging from completing fasting days
4. Social camouflage and disclosure dilemmas in managing identity and relationships	4a. Discreet management of diabetes equipment	Concealing insulin pens, pumps, and monitoring devices in social and professional settings
	4b. Timing medical activities around social situations	Scheduling injections, glucose checks, and snacking to avoid public observation
	4c. Cover stories for diabetes-related behaviors	Developing explanations for absences, dietary choices, or visible equipment that avoid full disclosure
	4d. Marriageability and romantic disclosure concerns	Culturally specific anxieties about revealing T1D to prospective partners and their families
	4e. Cost of concealment to management quality	Instances where prioritizing secrecy led to missed doses, delayed treatment, or poor glucose control
5. Resilience and cultural innovation for transforming challenge into strength	5a. Creative adaptation of cultural practices	Innovative modifications to celebrations, meals, exercise, and religious observances
	5b. Peer education and advocacy within communities	Voluntarily educating friends, family, and community members about T1D
	5c. Building supportive networks with other young adults	Forming or joining diabetes peer groups for mutual support and knowledge sharing
	5d. Reframing diabetes as strength and wisdom	Viewing the discipline and knowledge gained from T1D management as personal assets
	5e. Leadership in promoting cultural competence in healthcare	Advocating for healthcare providers to better understand cultural needs of Saudi patients

**Figure 1:**
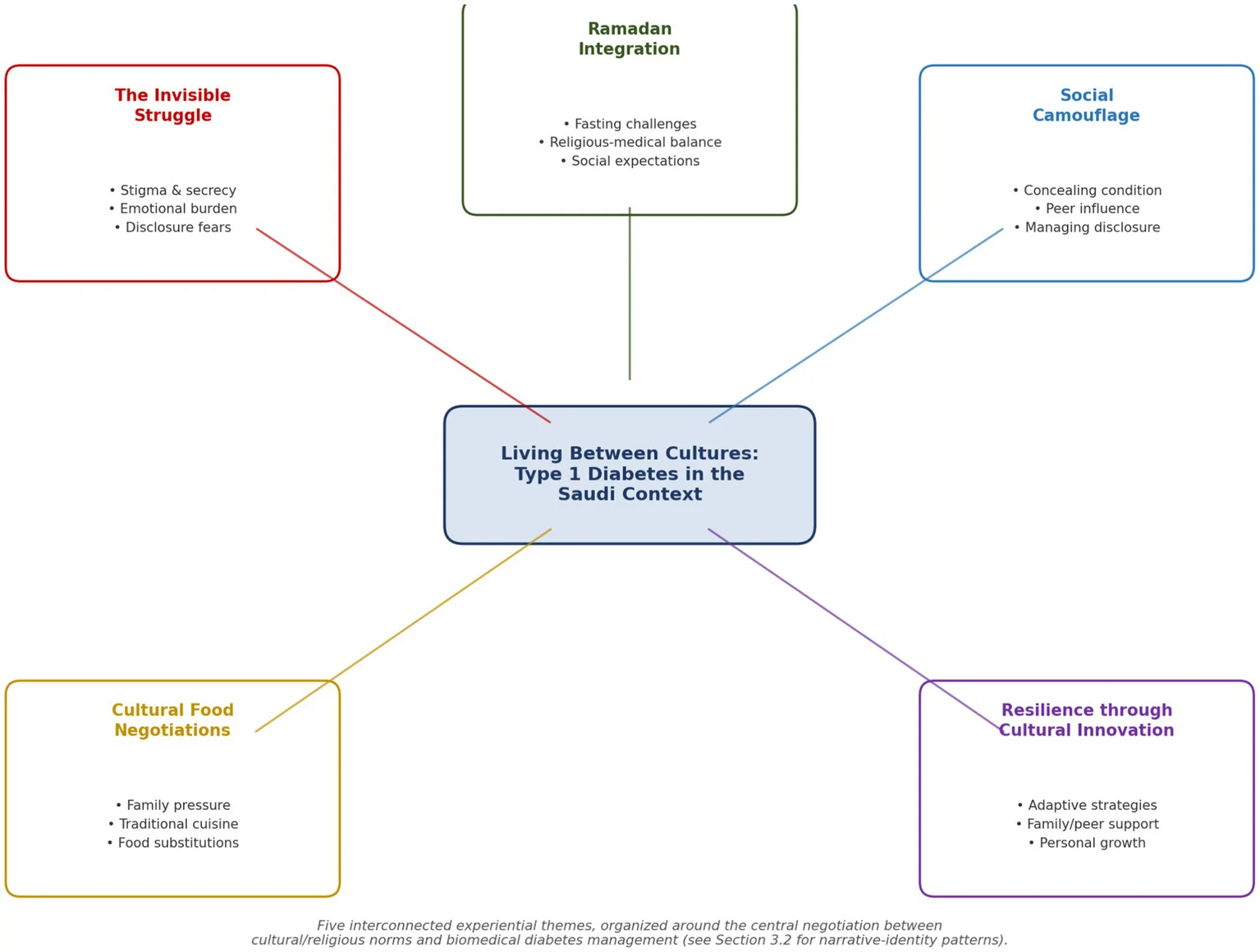
Multi-level thematic integration.

The five major themes and their interconnected relationships are illustrated in Figure 1. These themes were not discrete categories but rather dynamically interrelated dimensions of participants’ experiences navigating diabetes management within their cultural context. Each theme encompasses multiple subthemes that reflect specific challenges and adaptation strategies.

### 3.3 Theme 1: the invisible struggle in managing a hidden chronic condition

Participants consistently described T1D as an “invisible” condition that created ongoing challenges in gaining understanding and support from their social environments. Unlike visible disabilities or acute illnesses, T1D required constant management activities that were largely unobservable to others, leading to frequent misunderstandings and limited accommodation.

The invisibility of the condition created a persistent tension between appearing “normal” and meeting medical needs. Participants described elaborate strategies for concealing blood glucose monitoring, insulin administration, and dietary restrictions in social and professional settings. P2 explained:

"People see me as completely healthy and normal. They don't understand that I'm constantly calculating, monitoring, adjusting everything I do. Even when I'm laughing and enjoying myself at a gathering, part of my mind is always focused on my blood sugar levels and when I need to eat or take insulin."

This invisible struggle extended to the emotional burden of constant vigilance. Participants described the mental exhaustion of continuously monitoring their bodies, anticipating potential complications, and making complex decisions about food, activity, and insulin dosing throughout each day. P7 described this weight:

"Some nights I lie awake thinking: did I calculate correctly? Is my blood sugar going to drop while I sleep? My roommate at university has no idea I set alarms at 2 a.m. to check my glucose. He just thinks I'm a light sleeper."

The cognitive load of diabetes management was rarely acknowledged by others, contributing to feelings of isolation. P12, a medical student herself, offered a particularly apt observation:

"Even in my school, where everyone studies diabetes, my classmates don't truly grasp what it means to live with it every single minute. They memorize the pathophysiology, but they don't understand the exhaustion of being your own doctor, nurse, and patient all at once, 24 hours a day."

P5, who had lived with T1D for only 2 years, described a contrasting approach. Rather than concealing his condition, he chose visible management from the outset, injecting insulin openly in the university cafeteria and wearing his glucose monitor on his arm:

"I decided from day one I wasn't going to hide it. Some people stared, a few made comments, but most just got used to it. I think hiding would have made me feel more ashamed, like there was something wrong with me. There is nothing wrong with me."

P5’s approach, while atypical in this sample, shows that concealment was not universal and that individual coping styles and timing of diagnosis shaped disclosure behavior.

### 3.4 Theme 2: cultural food negotiations in balancing tradition and medical needs

Food emerged as the primary site of negotiation between cultural practices and medical requirements. Saudi culture places strong emphasis on hospitality, communal dining, and food as an expression of love and social connection. Participants described the complex emotions involved in navigating these food-centered practices.

Traditional Saudi dishes, often high in carbohydrates and prepared with substantial amounts of rice, dates, and sweetened ingredients, presented ongoing challenges for carbohydrate counting and insulin dosing. Participants developed detailed knowledge of traditional recipes to enable more accurate estimation, often working with family members to modify preparation methods. P4 shared:

“When my grandmother makes her special kabsa for family gatherings, she puts so much love and tradition into that dish. It’s not just food because it’s her way of caring for the family and maintaining our connections to our ancestors. When I have to be careful about portions or ask about ingredients, I can see the hurt in her eyes sometimes, even though she tries to be understanding.”

Family dynamics around food were particularly difficult to navigate. Refusing traditional dishes could be interpreted as a rejection of family love and cultural heritage. P3 described a collaborative approach:

“I started learning to cook with my mother. I would say, ‘Let me help you because I want to learn our family recipes.’ While cooking together, I could gently adjust ingredients by using less sugar, more vegetables, and brown rice instead of white. She saw it as me honoring her traditions, which I was, however I was also protecting my health.”

P6 highlighted the emotional complexity of communal dining:

"At large family gatherings, my aunts will pile food on my plate. 'Eat, eat, you're too thin!' they say. If I refuse, they look hurt or suspicious. I've learned to take small portions of everything and eat slowly, so it looks like I'm participating fully. But inside, I'm doing math throughout the entire meal, as I’m counting every grain of rice."

P9 described persistent family conflict around food that he had been unable to resolve:

"My father still doesn't understand. After 12 years, he tells me I'm having difficulty, that my grandfather ate dates every day and lived to be 90. He takes my portion control as a personal insult. We've had real fights about it. At some family meals, I just eat what's served and deal with the high blood sugar afterward because the argument isn't worth it."

P9’s account shows that cultural food negotiations were not always successful and that family resistance to dietary modification could lead to self-reported lapses in glycemic self-management (no clinical data were collected), with potential implications for family-inclusive care models.

### 3.5 Theme 3: Ramadan as a test of faith and health through spiritual and medical integration

Ramadan emerged as a particularly complex period requiring careful integration of spiritual commitments and medical requirements. All participants valued participation in Ramadan observances, yet the dramatic changes in eating patterns, sleep schedules, and daily routines created significant management challenges.

Participants described Ramadan as both a spiritual opportunity and a medical challenge, requiring extensive preparation and close collaboration with healthcare providers. P1 explained:

"Ramadan is the most spiritually important time of year for me. It's when I feel closest to Allah and most connected to my community. But it's also the most medically challenging time. I spend months preparing with my doctor, adjusting my insulin regimen, planning my meals, and establishing emergency protocols. It's like preparing for a marathon that lasts a month."

Preparation for Ramadan began weeks in advance: adjusting medication timing, developing modified meal plans for pre-dawn (suhoor) and sunset (iftar) meals, and establishing hypoglycemia protocols. P8 described the emotional significance of being able to participate alongside her family:

"When I sit with my family at iftar and we break our fast together, and I know my blood sugar has been stable all day, then it is one of the best feelings in my life. I feel normal. I feel strong. I feel like my diabetes doesn't define me."

P10 described the peer and family pressure she navigated:

"My cousins who don't know about my diabetes would ask, 'Why aren't you fasting today?' Even the ones who know would say, 'But you look fine, so can't you try?' There's a subtle pressure, which is not mean-spirited but remains constant. You feel like you're letting people down when you can't fast."

P11 described a frightening experience during his first Ramadan attempt after diagnosis:

"I insisted on fasting even though my doctor advised against it. I was only 16 and I wanted to be like everyone else. By the third day, I collapsed at the mosque. My blood sugar had dropped to 42. My father carried me out. I felt ashamed not because of the diabetes, but because I had put myself in danger for pride. I've finally made peace with that, but it took years."

P11’s account illustrates that social pressure to fast can lead to medically dangerous decisions, particularly among younger or recently diagnosed individuals, with direct implications for patient counseling and pre-Ramadan collaborative care planning.

### 3.6 Theme 4: social camouflage and disclosure dilemmas in managing identity and relationships

Participants developed sophisticated strategies for managing information about their diabetes in various social contexts, often engaging in what they described as “social camouflage” (25) to avoid unwanted attention, discrimination, or misunderstanding. The decision of when, how, and to whom to disclose their condition required constant negotiation.

In educational and professional settings, participants often concealed their condition to avoid being perceived as less capable. P14 described his workplace strategy:

“I excuse myself to the bathroom to inject insulin. I keep my supplies in a small pouch that looks like a toiletry bag. When colleagues ask why I always carry it, I say it’s hand sanitizer and personal items.”

P13 described the workplace cost of concealment:

“One day at work, I felt my blood sugar dropping but I was in the middle of a meeting with my manager. I did not want to pull out my glucose meter in front of everyone, so I waited. By the time the meeting ended, I was shaking and could barely stand. I had to sit in my car for 30 min eating candy. That’s when I realized hiding my diabetes could actually be dangerous.”

Marriage and romantic relationships presented culturally specific disclosure challenges. P3 shared:

“In my culture, when families are considering marriage arrangements, health is very important. I worry that having diabetes will make me less attractive as a potential husband. I know I can live a normal, healthy life, but I’m not sure other families will understand that. It affects how I think about marriage and when I should tell someone about my condition.”

P2 offered the female perspective on marriageability:

“My mother worries that when a family comes to ask about me for their son, they’ll hear ‘diabetes’ and leave. She once suggested I do not mention it until after engagement. I refused because I don’t want to start a marriage with a secret. But I understand her fear. In our culture, a girl’s health affects her family’s reputation.”

P8 described choosing full disclosure early in her relationship with her fiancé:

“I told them and I showed them my insulin pen, my glucose monitor, everything. His family had questions because they were worried about pregnancy complications.”

P8’s positive experience, while not representative of all participants’ fears, showed that disclosure does not invariably lead to rejection and that educational interventions targeting prospective families could help normalize T1D in marriage contexts.

### 3.7 Theme 5: resilience and cultural innovation for transforming challenge into strength

Despite the challenges documented in previous themes, participants showed genuine creativity in adapting cultural practices to accommodate medical needs while maintaining cultural identity. This theme captures the ways participants transformed their diabetes experience from a source of constraint into a platform for personal and community leadership.

P10 described how she became a cultural innovator in her family:

“I’ve learned to make traditional sweets using natural sweeteners for Eid celebrations. My family was skeptical at first, but now they request these versions because they taste so good and are healthier for everyone. I feel like I’m not just managing my diabetes but also helping my whole family eat better while keeping our traditions alive.”

Many participants became advocates and educators within their families and communities. P7 described his peer mentoring activities:

“I started a social account in Arabic where I share diabetes-friendly versions of Saudi recipes and tips for managing T1D during Ramadan. I have followers now, mostly other young Saudis with diabetes. When someone messages me saying, ‘Your recipe helped me enjoy Eid without a glucose spike,’ that makes everything worth it.”

P13 reflected on how diabetes had paradoxically become a source of personal pride:

“I used to ask, ‘Why me?’ Now I think diabetes gave me discipline, knowledge about health, and empathy for others who struggle. I’m stronger because of it. When I successfully manage my blood sugar through a full day of Ramadan fasting, I feel like I can do anything.”

P11, who was still in the early years of adjustment, provided a contrasting perspective:

“I hear people say diabetes made them stronger and I think, ‘Not yet for me.’ I’m still angry sometimes. I still feel like I lost something. Maybe I’ll get to that place eventually, but right now, I just want to get through a normal week without something going wrong.”

P11’s account is an important qualifier: the resilience narrative, while prominent among more experienced participants, was not universal. The trajectory toward positive reframing appeared to develop over time and was not accessible to all individuals at all stages.

### 3.8 Narrative patterns across participant stories

Beyond thematic content, three overarching narrative patterns emerged that characterized how participants structured and made meaning of their diabetes experiences. These patterns represented different ways of constructing identity and purpose in relation to chronic illness.

The Journey Narrative was the most common pattern (*n* = 7: P2, P4, P3, P6, P8, P13, P12), with participants describing their diabetes experience as an ongoing journey through distinct phases: initial shock and denial, followed by learning and adaptation, then acceptance and mastery, and ultimately advocacy and helping others. P6 captured this pattern:

“If I look back at the 15-year-old girl who cried every night after diagnosis and compare her to who I am now, as a teacher and a wife and someone who helps other women with diabetes, it’s like watching a character grow in a novel. Every chapter was necessary.”

The Warrior Narrative emerged among four participants (P1, P7, P5, P9) who framed their diabetes management as a daily battle requiring strength, vigilance, and determination. P1 exemplified this pattern:

“Every morning I wake up and I go to war with my blood sugar. Some days I win, some days it wins. But I never give up. My father taught me that a real man does not run from a fight, and this is the biggest fight of my life.”

The Bridge-Builder Narrative characterized three participants (P10, P12, P14) who saw themselves as bridges between traditional culture and modern medicine. P12 described:

“I want to be the kind of doctor who understands both worlds, including the world of medicine and the world of my patients’ culture. I’ve lived in both. That’s my purpose, which is to build a bridge so that no young Saudi person has to choose between their health and their identity.”

Overlap existed between patterns: several participants displayed characteristics of more than one narrative identity. P11 did not fit any positive narrative pattern, instead describing ongoing struggle without resolution, consistent with his shorter disease duration. The three narrative identity patterns identified in this study emerged inductively through an iterative process of engagement with participants’ narratives. During analysis, researchers examined how participants positioned themselves in relation to diabetes, family expectations, cultural values, and religious beliefs. Attention was given to narrative elements including turning points, coping strategies, identity negotiation, and future-oriented meanings. Initial codes were developed from repeated readings of transcripts, followed by the identification of broader narrative patterns. Through ongoing comparison across participants’ accounts, three overarching narrative identity patterns were developed: the Journey, Warrior, and Bridge-Builder narratives. These patterns were not predefined categories but represented recurring ways in which participants described and interpreted their diabetes experiences; they are interpretive constructions representing dominant patterns of meaning-making rather than mutually exclusive participant categories. Participant narratives were assigned to the dominant narrative pattern that best represented their overall meaning-making process. In cases where narratives reflected characteristics of multiple patterns, such as P12, researchers discussed the interpretation collectively and reached agreement regarding the primary narrative orientation. The final categorisation represented an interpretive synthesis rather than a rigid classification.

## 4 Discussion

### 4.1 Living between cultures as an acculturation-like negotiation

The findings reveal the sophisticated negotiation through which young Saudi adults with T1D move between traditional cultural practices and modern medical requirements. Consistent with Berry’s (9) acculturation framework, participants did not simply choose between cultural practices and medical requirements but engaged in ongoing negotiations that transformed both domains. Just as individuals navigating cultural transitions may develop integration strategies drawing on both heritage and new-context resources, these participants developed hybrid practices that honored cultural identity while meeting health needs. As noted in the Introduction, Berry’s framework is invoked here analogically rather than literally: these participants were not navigating migration-related acculturation, and the parallel should be read as illustrative of a structurally similar negotiation between normative systems, not as evidence that the same mechanisms are at work. The framework was used as an interpretive lens to understand how participants negotiated multiple cultural influences shaping diabetes self-management, and we acknowledge that it represents one of several possible theoretical perspectives rather than the sole explanation for participants’ experiences.

The negotiation strategies participants employed evolved over time. Early post-diagnosis phases involved rigid compartmentalization, with medical and cultural domains kept largely separate. Later phases showed more sophisticated integration strategies. This temporal evolution was particularly evident among participants with longer disease duration (P2, P3, P6, P10), whose narratives described progressive mastery, and less evident among recently diagnosed participants (P5, P11).

These findings challenge deficit-based healthcare models that frame cultural practices as “barriers” to overcome (26). Participants’ capacity to maintain cultural engagement alongside T1D management suggests that culturally responsive care should facilitate integration rather than demand cultural concession.

### 4.2 Family systems as double-edged resources

Consistent with scholarship on chronic illness management in collectivistic societies (16), family involvement emerged as both a primary resource and a significant stressor. Participants who successfully engaged families in diabetes education, such as through P3’s collaborative cooking approach and P10’s recipe innovations, reported greater satisfaction and better perceived management, aligning with family systems approaches to chronic illness (27). However, the family-centered nature of Saudi culture also constrained autonomy, as illustrated by P9’s unresolved food conflicts and P2’s mother’s suggestion to conceal the diagnosis from prospective marriage partners.

This dual role complicates the emphasis on patient autonomy prevalent in Western diabetes care models and supports recommendations for family-inclusive education programs tailored to collectivistic contexts. Healthcare providers in these settings require training in family systems approaches that respect cultural values while promoting effective diabetes management.

### 4.3 Spiritual integration, not spiritual conflict

The prominence of Ramadan in participants’ narratives extends existing clinical research on Ramadan and diabetes (13, 14) by foregrounding the experiential and spiritual dimensions of fasting decisions. Participants did not frame Ramadan as a medical obstacle but as an identity-defining practice demanding creative clinical collaboration. P1’s “marathon” metaphor and P8’s description of emotional fulfillment at iftar demonstrate that successful fasting enhanced community belonging and management motivation. P11’s collapse at the mosque, by contrast, illustrates the real medical dangers when social pressure overrides clinical judgment, with direct implications for patient counseling.

Healthcare providers working with Muslim patients with T1D need training that extends beyond clinical protocols to encompass understanding of the spiritual significance of religious participation and collaborative planning approaches that respect religious commitment while establishing clear safety boundaries (28).

### 4.4 Social camouflage and chronic illness stigma

The “social camouflage” strategies documented in Theme 4 mirror Goffman’s (25) and subsequent work on concealment in chronic illness (29). Concealment, while protective against discrimination, carried costs: P13’s near-emergency during a meeting and the persistent psychological burden described by P14 illustrate how prioritizing secrecy could compromise both safety and well-being. The culturally specific marriageability concerns expressed by both P3 and P2 add a dimension not well captured in Western chronic illness literature, where marriage is typically an individual rather than a family-level decision.

These findings support targeted public education distinguishing T1D from Type 2 diabetes and legal protections for individuals with chronic conditions in educational and employment settings. The positive outcome of P8’s early disclosure to her fiancé suggests that educational interventions could also target prospective families, normalizing T1D within the marriage context.

### 4.5 Resilience as an ecologically embedded process

The cultural innovations developed by participants, including modified recipes, adapted exercise routines, integrated religious-medical practices, and peer education platforms, represent what Ungar (30) terms “culturally and contextually relevant” resilience processes. Rather than emerging from individual traits alone, this resilience was ecologically embedded in family support, healthcare partnerships, and community belonging. However, P11’s candid account is an important qualifier: resilience narratives must not be imposed prescriptively, particularly on individuals in early adjustment phases, for whom ongoing struggle represents a normative response warranting targeted psychosocial support.

Healthcare systems should develop mechanisms for documenting and disseminating patient-generated innovations. Peer mentorship programs that build on the natural leadership demonstrated by P7’s social media advocacy and P12’s career aspirations could connect newly diagnosed individuals with experienced peers in structured, clinically supported settings.

### 4.6 Limitations

Several limitations should be noted. Recruitment from urban centers limits transferability to rural Saudi communities, where healthcare access, traditional practices, and social dynamics may differ substantially. Individuals living in rural or geographically remote areas may experience different challenges related to healthcare access, family structures, social expectations, and available support systems. Furthermore, while some cultural and religious influences may resonate with young adults across Gulf and Middle Eastern contexts, differences in social norms, healthcare systems, and community practices should be considered; transferability of the findings should therefore be assessed according to contextual similarity rather than statistical generalisation. Voluntary participation may have introduced self-selection bias, potentially over-representing individuals with more positive adaptation experiences. The cross-sectional design captures experiences at a single time point; longitudinal designs would better illuminate how negotiation strategies evolve across the disease trajectory. All interviews were conducted by a single researcher, ensuring consistency but potentially introducing interviewer effects. No clinical outcome measures (e.g., HbA1c) were collected, so assessments of glycemic management quality rely entirely on participant self-report. Although a forward-backward translation protocol was employed, idiomatic nuances of Arabic expression may not have been fully preserved. Finally, while 14 participants is appropriate for qualitative narrative inquiry and achieved thematic saturation, the breadth of demographic variation is necessarily limited. Recall bias is an inherent limitation of retrospective narrative accounts; participants’ descriptions of earlier disease phases may reflect current meaning-making frameworks rather than contemporaneous experience. Social desirability bias cannot be excluded, as participants may have emphasised positive adaptation strategies when speaking with a healthcare professional. The sample was recruited exclusively through urban professional and university networks, limiting representativeness among Saudi adults with lower educational attainment or limited healthcare engagement. Participants’ self-reported glycemic management should not be conflated with clinical outcomes; the absence of HbA1c data means the relationship between the cultural strategies described and objectively measured glucose control remains untested. Future studies integrating clinical biomarkers with qualitative inquiry would significantly strengthen the evidence base for culturally responsive diabetes care in this population.

## 5 Conclusion

Young Saudi adults with T1D exercise genuine cultural agency in navigating the intersection of traditional practices and biomedical management. Rather than experiencing culture and medical management as irreconcilably opposed, participants engaged in sophisticated, temporally evolving negotiation processes that transformed both their cultural practices and their approach to diabetes care.

Five interconnected experiential dimensions, namely the invisible struggle of managing a hidden condition, food-centered cultural negotiations, Ramadan as a site of spiritual and medical integration, social camouflage and disclosure dilemmas, and resilience through cultural innovation, illuminate chronic illness management as a fundamentally cultural process. Three narrative identity patterns (Journey, Warrior, Bridge-Builder) further reveal the active meaning-making through which participants construct coherent identities in the context of chronic illness. Deviant cases, including sustained family resistance, medically dangerous fasting attempts, and persistent concealment that participants themselves linked to worse glycemic self-management, demonstrate that these processes are neither universally positive nor linear.

These findings carry substantive implications for healthcare delivery. Culturally responsive care models should treat patients’ cultural knowledge as a clinical resource, actively engage families as management partners, and provide targeted, collaborative support for religious observance, including individualized, pre-Ramadan risk assessment and shared decision-making between patients and healthcare professionals, taking into account each person’s clinical status, risk profile, and preferences in accordance with current diabetes management guidelines, rather than uniform fasting guidance, given how differently participants such as P11 and P8 fared. Peer mentorship programs leveraging experienced patients’ cultural innovations, and family-inclusive diabetes education adapted to collectivistic contexts, represent priority intervention targets. Future research should integrate clinical outcome measures, extend inquiry to rural and Gulf Cooperation Council populations, employ longitudinal designs, and rigorously evaluate culturally adapted education programs.

## Data Availability

Data are available within the article and Supplementary materials. Raw interview transcripts are not publicly available to protect participant confidentiality but may be made available to qualified researchers on reasonable request to the corresponding author, subject to institutional data governance review.
