# Supplementary material for: Navigating tradition and treatment: cultural identity, social expectations, and type 1 diabetes self-management among young Saudi adults–a qualitative narrative inquiry
Source: Front Public Health. 2026 Aug 31;14:1920664. doi: 10.3389/fpubh.2026.1920664 (PMC13572735; doi:10.3389/fpubh.2026.1920664)
Supplement: Supplementary file 1 [file Table_1.DOCX]

**Supplementary File S1**

**COREQ Checklist - Consolidated Criteria for Reporting Qualitative Research**

Reference: Tong A, Sainsbury P, Craig J. Consolidated criteria for reporting qualitative research (COREQ): a 32-item checklist for interviews and focus groups. Int J Qual Health Care. 2007;19(6):349–357. https://doi.org/10.1093/intqhc/mzm042

This checklist covers all 32 COREQ items across three domains. All applicable items are addressed below and cross-referenced to the corresponding section of the manuscript. ‘N/A’ indicates items not applicable to this study design.

| **Domain / Sub-domain** | **No.** | **Topic** | **Item** | **Location in Manuscript / Note** | **Reported** |
| --- | --- | --- | --- | --- | --- |
| **DOMAIN 1: Research Team and Reflexivity** | | | | | |
| **1a. Personal Characteristics** | | | | | |
| Personal characteristics | **1** | **Interviewer / facilitator** | Which author(s) conducted the interview or focus group? | Lead researcher (A.M.A.) conducted all 14 interviews. See Section 2.4. | **Yes** |
|  | **2** | **Credentials** | What were the researcher's credentials, e.g. PhD, MD? | Doctoral in nursing (A.M.A.). See Section 2.4. | **Yes** |
|  | **3** | **Occupation** | What was their occupation at the time of the study? | Doctoral researcher and lecturer in nursing at King Saud University. See Section 2.4. | **Yes** |
|  | **4** | **Gender** | Was the researcher male or female? | Male. Not explicitly stated in the manuscript to avoid participant identification; available on request. | **Yes** |
|  | **5** | **Experience and training** | What experience or training did the researcher have? | Clinical experience in diabetes care; formal training in qualitative interviewing and chronic condition research. See Section 2.4. | **Yes** |
| **1b. Relationship with Participants** | | | | | |
| Relationship with participants | **6** | **Relationship established** | Was a relationship established prior to study commencement? | No prior clinical or personal relationship with any participant. This was explicitly confirmed during recruitment. See Section 2.4. | **Yes** |
|  | **7** | **Participant knowledge of the interviewer** | What did the participants know about the researcher, e.g. personal goals, reasons for doing the research? | Participants were informed of the researcher's role, institutional affiliation, study purpose, and sponsorship during the consent process. See Section 2.3. | **Yes** |
|  | **8** | **Interviewer characteristics** | What characteristics may have influenced the interview, e.g. bias, assumptions, reasons and interests in the research topic? | The researcher's clinical diabetes background and Muslim identity were acknowledged as potential assets and sources of bias. Reflexivity was maintained through analytical journaling. See Section 2.7. | **Yes** |
| **DOMAIN 2: Study Design** | | | | | |
| **2a. Theoretical Framework** | | | | | |
| Theoretical framework | **9** | **Methodological orientation and theory** | What methodological orientation was stated to underpin the study, e.g. grounded theory, discourse analysis, ethnography, phenomenology, content analysis? | Narrative inquiry (Clandinin & Connelly, 2000) [22]; cultural psychology (Bruner, 2002) [24] as primary theoretical framework; Berry's (1997) [9] acculturation model applied analogically as secondary conceptual lens. See Section 2.1. | **Yes** |
| **2b. Participant Selection** | | | | | |
| Participant selection | **10** | **Sampling** | How were participants selected, e.g. purposive, convenience, consecutive, snowball? | Purposive sampling supplemented by snowball sampling. Rationale: to capture diverse experiences while maintaining focus on the research question. See Section 2.2. | **Yes** |
|  | **11** | **Method of approach** | How were participants approached, e.g. face-to-face, telephone, mail, email? | Via professional networks, family connections, and university student services; subsequent participants referred by early recruits (snowball). See Section 2.2. | **Yes** |
|  | **12** | **Sample size** | How many participants were in the study? | 14 participants (8 male [57.1%], 6 female [42.9%]). See Section 2.2 and Table 1. | **Yes** |
|  | **13** | **Non-participation** | How many people refused to participate or dropped out? Reasons? | No dropouts occurred after written informed consent was provided. Not reported in the main manuscript; available in the study records. | **Yes** |
| **2c. Setting** | | | | | |
| Setting | **14** | **Setting of data collection** | Where was the data collected, e.g. home, clinic, workplace? | University offices, private hospital rooms, and participants' homes, all chosen by participants to ensure comfort and privacy. See Section 2.4. | **Yes** |
|  | **15** | **Presence of non-participants** | Was anyone else present besides the participants and researchers? | No. Interviews were conducted with only the lead researcher and the participant present. See Section 2.4. | **Yes** |
|  | **16** | **Description of sample** | What are the important characteristics of the sample, e.g. demographic data, date? | Full demographic profile (age, gender, T1D duration, insulin regimen, glucose monitoring, Ramadan fasting, education, employment, marital status) presented in Table 1. Data collected October 2024 – January 2025. | **Yes** |
| **2d. Data Collection** | | | | | |
| Data collection | **17** | **Interview guide** | Were questions, prompts, guides provided by the authors? Was it pilot tested? | 15 open-ended questions developed following a systematic literature review and refined after pilot testing with two individuals not included in the final sample. Full guide provided in Appendix A. | **Yes** |
|  | **18** | **Repeat interviews** | Were repeat interviews carried out? If yes, how many? | Follow-up contact was made with 5 participants for member-checking purposes only. No repeat full interviews were conducted. See Section 2.7. | **Yes** |
|  | **19** | **Audio/visual recording** | Did the research use audio or visual recording to collect the data? | All interviews were audio-recorded on encrypted digital devices with explicit participant permission. See Section 2.4. | **Yes** |
|  | **20** | **Field notes** | Were field notes made during and/or after the interview? | Detailed field notes were maintained throughout data collection, documenting contextual information, nonverbal observations, and preliminary analytical reflections. See Section 2.4. | **Yes** |
|  | **21** | **Duration** | What was the duration of the interviews or focus group? | Ranged from 45 to 90 minutes (mean = 67 min). Duration was determined by response richness and natural narrative conclusion. See Section 2.4. | **Yes** |
|  | **22** | **Data saturation** | Was data saturation discussed? | Yes. Thematic saturation was assessed after interview 12; interviews 13 and 14 confirmed saturation, with no new themes or substantial variation emerging. See Section 2.4. | **Yes** |
|  | **23** | **Transcripts returned** | Were transcripts returned to participants for comment and/or correction? | Theme summaries (not verbatim transcripts) were returned to 5 participants for member checking. Participants confirmed accuracy and provided minor clarifications incorporated into analysis. See Section 2.7. | **Yes** |
| **DOMAIN 3: Analysis and Findings** | | | | | |
| **3a. Data Analysis** | | | | | |
| Data analysis | **24** | **Number of data coders** | How many data coders coded the data? | Primary coding was conducted by the lead researcher (A.M.A.). All co-authors participated in theme review and discussion. See Section 2.6. | **Yes** |
|  | **25** | **Description of the coding tree** | Did authors provide a description of the coding tree? | Yes. The coding framework (themes, subthemes, and representative codes) is available as Supplementary Appendix B. See Section 2.6. | **Yes** |
|  | **26** | **Derivation of themes** | Were themes identified in advance or derived from the data? | Themes were derived inductively from the data, consistent with Braun and Clarke's (2019) reflexive thematic analysis approach. See Section 2.6. | **Yes** |
|  | **27** | **Software** | What software, if applicable, was used to manage the data? | Analysis was conducted manually. An audit trail of analytical decisions, memos, and theme development was maintained throughout. See Section 2.6. | **N/A** |
|  | **28** | **Participant checking** | Did participants provide feedback on the findings? | Yes. Member checking was conducted with five participants who reviewed theme summaries and confirmed interpretive accuracy. Minor clarifications were incorporated. See Section 2.7. | **Yes** |
| **3b. Reporting** | | | | | |
| Reporting | **29** | **Quotations presented** | Were participant quotations presented to illustrate the themes / findings? Was each quotation identified? | Yes. Participant quotations are used throughout Sections 3.3–3.8 to illustrate all five themes and all three narrative identity patterns. Each quotation is identified by participant pseudonym (P1–P14). | **Yes** |
|  | **30** | **Data and findings consistent** | Was there consistency between the data presented and the findings? | Yes. All themes and sub-themes are illustrated with multiple participant quotations. Deviant cases that challenge dominant patterns are explicitly identified and analysed across all five themes. See Sections 3.3–3.7. | **Yes** |
|  | **31** | **Clarity of major themes** | Were major themes clearly presented in the findings? | Yes. Five major themes and their sub-themes are presented in Sections 3.3–3.7 and summarised with descriptions in Table 2. An overarching thematic model is described in Figure 1. | **Yes** |
|  | **32** | **Clarity of minor themes** | Is there a description of diverse cases or discussion of minor themes? | Yes. Deviant and negative cases are systematically integrated across all five themes. Three narrative identity patterns (Journey, Warrior, Bridge-Builder) are presented in Section 3.8 as a higher-order analytic layer cutting across themes. | **Yes** |
