# Supplementary material for: Navigating tradition and treatment: cultural identity, social expectations, and type 1 diabetes self-management among young Saudi adults–a qualitative narrative inquiry
Source: Front Public Health. 2026 Aug 31;14:1920664. doi: 10.3389/fpubh.2026.1920664 (PMC13572735; doi:10.3389/fpubh.2026.1920664)
Supplement: Supplementary file 2 [file Table_2.DOCX]

**Appendix A**

**Semi-Structured Interview Guide**

The guide was pilot tested with two individuals who met all inclusion criteria but were not included in the final sample. Following pilot testing, question wording in Sections 3 and 4 was refined to reduce leading language, and additional probes were added to Section 5 in response to pilot participant feedback that questions about marriage felt abrupt without prior contextualisation.

Thank you very much for agreeing to speak with me today. As I explained during the consent process, the purpose of this study is to understand how young Saudi adults with Type 1 diabetes navigate the intersection of their cultural life and their diabetes management. I am interested in your personal story and experiences. There are no right or wrong answers, only your perspective matters. Please feel free to share as much or as little as you are comfortable with, and to stop the interview at any time without any consequence for you. With your permission, I will be audio-recording our conversation. Do you have any questions before we begin?

Section 1: Opening and Rapport-Building

Rationale: These opening questions are designed to establish rapport, set a narrative tone, and orient participants to the storytelling nature of the interview. They invite participants to introduce themselves and describe their daily lives in their own terms, without leading them toward specific themes.

Q1	Could you start by telling me a little about yourself and how long you have been living with Type 1 diabetes?

• How old were you when you were first diagnosed?

• How would you describe your first few weeks or months after diagnosis?

• Who was with you when you found out, and how did they react?

Q2	How would you describe your daily life managing diabetes, what does a typical day look like for you from the moment you wake up?

• Are there particular moments in the day that are more demanding or stressful than others?

• How does your management routine change depending on what you have planned?

Section 2: Cultural Identity and Daily Diabetes Management

Rationale: This section explores the intersection of Saudi cultural identity and T1D management. Questions are open-ended to allow participants to define what ‘culture’ means to them and to identify the dimensions they find most relevant, rather than imposing researcher-defined categories.

Q3	How do you feel your Saudi identity, cultural values, and background influence the way you manage your diabetes?

• Can you give me a specific example of a time when your cultural background affected a decision you made about your health?

• Are there particular values or expectations that make diabetes management easier or harder?

• How has this changed as you have gotten older?

Q4	Are there aspects of Saudi culture or traditional practices that you find particularly difficult to navigate because of your diabetes? Please tell me about those experiences.

• What strategies have you developed to manage these situations?

• Is there anyone who helps you navigate these challenges?

• Have you ever had to choose between following a cultural expectation and taking care of your health? What happened?

Q5	How do you balance the expectations of your family and community with the medical requirements of managing your diabetes?

• Has this balance changed over time?

• What do you wish the people around you understood better about your condition?

• Are there times when your family’s expectations make it harder to look after yourself?

Section 3: Food, Hospitality, and Social Gatherings

Rationale: Food is central to Saudi cultural identity and social life. This section explores how participants navigate the culturally significant practice of communal dining and hospitality within the constraints of T1D management, and how they have adapted traditional food practices.

Q6	Saudi culture places great importance on food, hospitality, and sharing meals together. How has having diabetes affected your experience of family meals, celebrations, and social gatherings?

• How do family members or hosts typically respond when you manage your diet differently from others?

• Have you ever felt embarrassed, excluded, or pressured in these situations?

• How do you handle situations where you are expected to eat things that are not suitable for you?

Q7	Have you found ways to adapt traditional foods, recipes, or cooking methods to better suit your diabetes management? If so, could you describe some of those adaptations?

• How did your family or friends respond to these changes?

• Have others in your family adopted any of these changes?

• Do you feel you have lost something important, or gained something, through these adaptations?

Section 4: Ramadan and Religious Observance

Rationale: Ramadan represents a uniquely complex intersection of religious obligation and medical management for Muslims with T1D. This section foregrounds the spiritual significance of Ramadan before addressing medical considerations, to avoid framing the religious practice as primarily a medical problem.

Q8	Ramadan is a central and deeply meaningful part of life for many Saudi Muslims. How do you experience Ramadan with Type 1 diabetes?

• Do you fast? If yes, how do you prepare medically and spiritually? If no, how do you navigate that decision within your family and community?

• What does participating in Ramadan, or not being able to participate fully, mean to you personally?

• Has your approach to Ramadan changed since your diagnosis?

Q9	Can you tell me about any specific experiences during Ramadan that have stayed with you, either because they were challenging, meaningful, or both?

• How did you feel emotionally and spiritually during that experience?

• How did the people around you respond?

• What did you learn from that experience?

Q10	How have your healthcare providers supported you, or not, in navigating Ramadan with diabetes?

• What kind of support would have been most helpful?

• Have you ever received advice that felt at odds with your religious values or community expectations?

• What would ideal support from a healthcare provider look like during Ramadan?

Section 5: Social Identity, Disclosure, and Stigma

Rationale: This section explores the social dimensions of living with T1D, including decisions about disclosure, experiences of stigma, and the culturally specific concern about how diabetes may affect personal and family reputation in the context of marriage and social standing.

Q11	How do you decide when and with whom to share information about your diabetes, for example, with friends, colleagues, university classmates, or in social situations?

• Have there been times when you chose to hide or minimise your diabetes? What led to that decision?

• What has happened when you have disclosed your condition? Were people supportive, or did they react in unexpected ways?

• Has managing information about your diabetes ever affected your safety or health?

Q12	How has having diabetes affected your relationships, including close friendships, romantic relationships, or thoughts about marriage and family?

• Have you experienced any negative reactions related to your diabetes in the context of relationships or marriage discussions?

• How do you decide when and how to disclose your condition to a prospective partner or their family?

• How has this affected how you see yourself and your future?

Q13	Have you ever felt judged, stigmatised, or treated differently because of your diabetes? Could you describe that experience?

• How did it affect you emotionally?

• How did you respond to it?

• Has that experience changed how you manage your diabetes publicly?

Section 6: Resilience, Adaptation, and Meaning-Making

Rationale: The final section invites participants to reflect on the broader meaning of their diabetes experience, including sources of strength and the ways in which they have adapted, innovated, or contributed to their communities. Questions are phrased to be open to both positive and negative experiences, to avoid prompting artificially optimistic responses.

Q14	Looking back over your experience of living with diabetes, what have been your most important sources of strength or support?

• Have you found ways to use your experience to help others, or to contribute to your family or community?

• Have you made any adaptations, to food, activities, or routines, that you are particularly proud of?

• Is there something that has surprised you about your own capacity to cope?

Q15	How has living with Type 1 diabetes shaped who you are, how you see yourself, or how you see the world?

• Is there anything positive that has come from this experience that you would like to share?

• Is there anything you feel you have lost or that remains difficult to accept?

• If you could say one thing to a young Saudi person who was just diagnosed with T1D, what would it be?

Closing Statement

Thank you very much for sharing your story and experiences with me today. Your account is important and will contribute meaningfully to improving healthcare for young people with Type 1 diabetes in Saudi Arabia. Before we finish, is there anything else you would like to add, or anything you felt was not covered that you would like to share?
